# Cardiac Masses: The Role of Cardiovascular Imaging in the Differential Diagnosis

**DOI:** 10.3390/diagnostics10121088

**Published:** 2020-12-14

**Authors:** Constantina Aggeli, Yannis Dimitroglou, Leonidas Raftopoulos, Georgia Sarri, Sophie Mavrogeni, Joyce Wong, Eleftherios Tsiamis, Costas Tsioufis

**Affiliations:** 1 First Department of Cardiology, General Hospital of Athens Hippokration, University of Athens Medical School, 11527 Athens, Attica, Greece; dimiyann@hotmail.com (Y.D.); lraftop@otenet.gr (L.R.); georgiasarri@hotmail.com (G.S.); ltsiamis@otenet.gr (E.T.); ktsioufis@hippocratio.gr (C.T.); 2 Department of Cardiology, Onassis Cardiac Surgery Centre, 17674 Kallithea, Attica, Greece; sophie.mavrogeni@gmail.com; 3 Department of Cardiology, Harefield Hospital and Royal Brompton Hospital, London UB96JH, UK; j.wong@rbht.nhs.uk

**Keywords:** cardiac tumors, cardiac malignancies, echocardiography, transesophageal echocardiography, contrast agents, cardiac magnetic resonance, cardiac computed tomography

## Abstract

Cardiac masses are space occupying lesions within the cardiac cavities or adjacent to the pericardium. They include frequently diagnosed clinical entities such as clots and vegetations, common benign tumors such as myxomas and papillary fibroelastomas and uncommon benign or malignant primary or metastatic tumors. Given their diversity, there are no guidelines or consensus statements regarding the best diagnostic or therapeutic approach. In the past, diagnosis used to be made by the histological specimens after surgery or during the post-mortem examination. Nevertheless, evolution and increased availability of cardiovascular imaging modalities has enabled better characterization of the masses and the surrounding tissue. Transthoracic echocardiography using contrast agents can evaluate the location, the morphology and the perfusion of the mass as well as its hemodynamic effect. Transesophageal echocardiography has increased spatial and temporal resolution; hence it is superior in depicting small highly mobile masses. Cardiac magnetic resonance and cardiac computed tomography are complementary providing tissue characterization. The scope of this review is to present the role of cardiovascular imaging in the differential diagnosis of cardiac masses and to propose a step-wise diagnostic algorithm, taking into account the epidemiology and clinical presentation of the cardiac masses, as well as the availability and the incremental value of each imaging modality.

## 1. Introduction

The diagnosis of space occupying lesions or masses in the heart can be done either in the context of investigation of a specific clinical symptomatology or incidentally, usually in the context of a cardiac imaging study performed for another reason. Cardiac masses can be classified as lesions that resemble tumors (clots, vegetations, calcifications or other rare lesions) and as benign or malignant, primary or metastatic, intracardial, or pericardial tumors [1,2,3]. Primary tumors are usually benign, with more than 50% chance of being a myxoma. Primary malignant tumors are rare, accounting for less than a quarter of primary heart tumors, in the majority of cases being sarcomas [4,5]. However, in autopsy studies a cardiac tumor most commonly represents a metastatic malignant tumor. The prevalence of primary cardiac tumors is 1:2000, while of metastatic 1:100 autopsies, reflecting a ratio of secondary to primary patients in the range of 20:1. The incidence of cardiac metastases varies among autopsy studies from 2.3% to 18.3% in patients with extracardiac malignant tumors [1,2,6].

Cardiac imaging modalities, including transthoracic (TTE) or transesophageal (TEE) echocardiography, cardiac magnetic resonance (CMR), cardiac computed tomography (CT), and ^18^Ffluorodeoxyglucose positron emission tomography (^18^F FDG-PET), have a complementary and reinforcing role for the evaluation of cardiac masses [7]. Given the diversity of cardiac masses, (from clots or vegetations to primary or metastatic malignant tumors), there are no guidelines or consensus statements regarding the best diagnostic or therapeutic approach. The scope of this review is to present the role of cardiovascular imaging in the differential diagnosis of cardiac masses and to propose a step-wise diagnostic algorithm, taking into account their epidemiology and clinical presentation, as well as the availability and the incremental value of each imaging modality.

## 2. Diagnostic Approach of Patients Presenting with Cardiac Masses

Evaluation begins with patient history and clinical examination including electrocardiogram followed by imaging data regarding size, localization, hemodynamic effects, vascularity, tissue characterization, and possible infiltration or compression of surrounding tissue. Clinicians should be familiar with epidemiological, clinical, and imaging features of most common cardiac masses (Table 1). Comprehensive presentation of those features is beyond the scope of the present review.

Knowledge of epidemiological data regarding frequency of each tumor, in the various age groups or between sexes and identification of accompanying symptoms and signs improves diagnostic accuracy of imaging tools.

**Figure 1:**
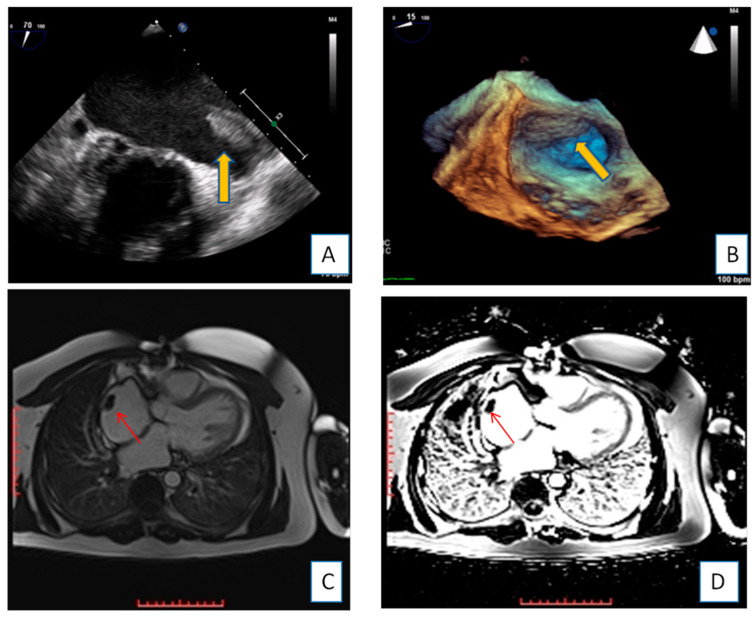
Transesophageal echocardiography depicting the left atrial appendage using 2D and 3D approach ((**A**,**B**), respectively). A huge thrombus (arrows) is demonstrated in the upper part of the “coumadin ridge” is an elderly woman with severe mitral stenosis. Clot in the RA (red arrows). Cine and EGE imaging ((**C**,**D**) respectively).

**Figure 2:**
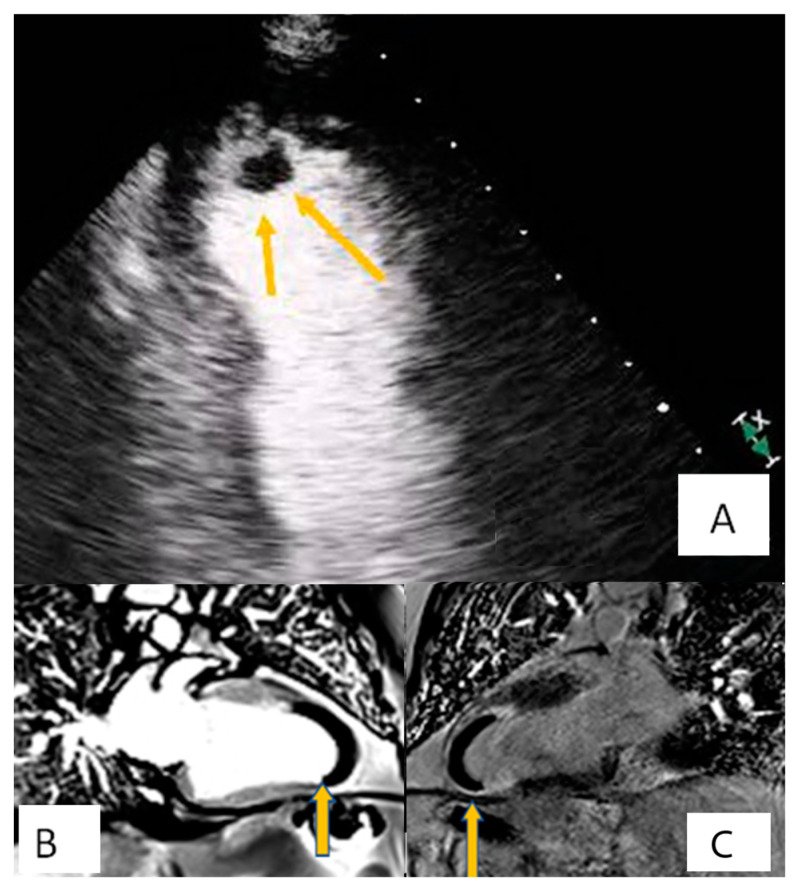
Patient presenting with anterior myocardial infarction the second day after primary percutaneous transluminar coronary angioplasty (PTCA). Apical 4-chamber view. (**A**) A lesion is depicted on cardiac apex (yellow arrows), mobile, without vasculature, findings compatible with clot. (**B**,**C**) Large laminar LV thrombus adjacent to an area of apical myocardial infarction. Two chambers EGE (image (**B**)) and LGE (image (**C**)) respectively.

The most frequent masses in the heart are clots or vegetations frequently accompanying a relevant clinical scenario such as mitral stenosis, atrial fibrillation, myocardial infarction, myocarditis with a very low ejection fraction, or infective endocarditis, respectively (Figure 1 and Figure 2).

Approximately 90% of the surgically-removed tumors are benign [4]. Most of these benign tumors are myxomas (80%), while the remaining ones are (in descending order) papillary fibroelastomas, fibromas, lipomas, and, even more rarely, calcified amorphous tumors, hemangiomas, teratomas, single developmental cysts, and rhabdomyomas [8,9,10,11]. Only about 10% of the primary cardiac tumors surgically removed are malignant: 90% of them identified as sarcomas or lymphomas [11].

On the other hand, secondary cancer metastases in myocardium and pericardium are more common. Secondary metastases can occur due to direct invasion of the primary tumor to the adjacent heart tissue (i.e., in case of breast or lung cancer) or hematogenous (arterial/venous) and lymphoid dispersion [12,13,14]. In certain tumor types, a genetic predisposition has been identified [15].

In infants and children, rhabdomyoma is the most frequent tumor type, followed by myxoma and fibroma [16,17]. Primary malignant tumors (usually rhabdomyosarcoma and teratoma) are very rare.

**Figure 3:**
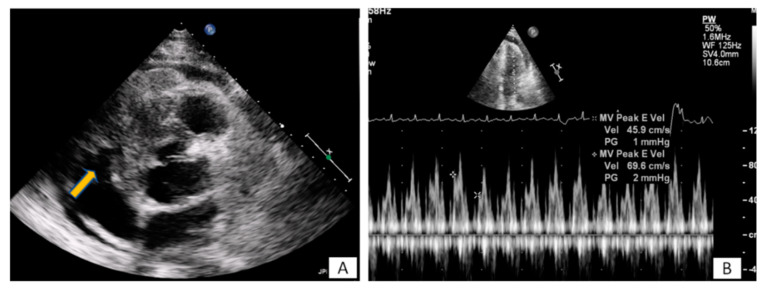
(**A**) A 65-year-old patient with fever and dyspnea for 15 days. Transthoracic echo (modified short axis view) demonstrated an amorphous mass (arrow) infiltrating the tricuspid annulus, right ventricular free wall and pericardium, causing a moderate in size pericardial effusion. (**B**) The pericardial fluid led to tamponade (**B**). Biopsy revealed a lymphoma.

Clinical symptoms and signs usually depend on the location of the tumor rather than its histological type [18,19]. Primary benign tumors as well as secondary malignant tumors may cause myocardial or valve dysfunction and could be accompanied by heart failure symptoms (most commonly dyspnea), angina, syncope and electrical disturbances of the heart or even fatal arrhythmias [20,21]. Pericardial effusion with or without tamponade is characteristic of malignant tumors (Figure 3). Embolism due to tumor particles is a relatively frequent complication, occurring in a quarter of all cardiac tumor patients, and it is related to anatomical and histological characteristics of each tumor. Constitutional manifestations such as weight loss, malaise, and fatigue have also been described. However, most cardiac masses remain asymptomatic or present with mild and atypical symptoms [1,2,3]. Hence, they are usually recognized during outpatient care.

## 3. Information Extracted by the Use of the Imaging Modalities

**Figure 4:**
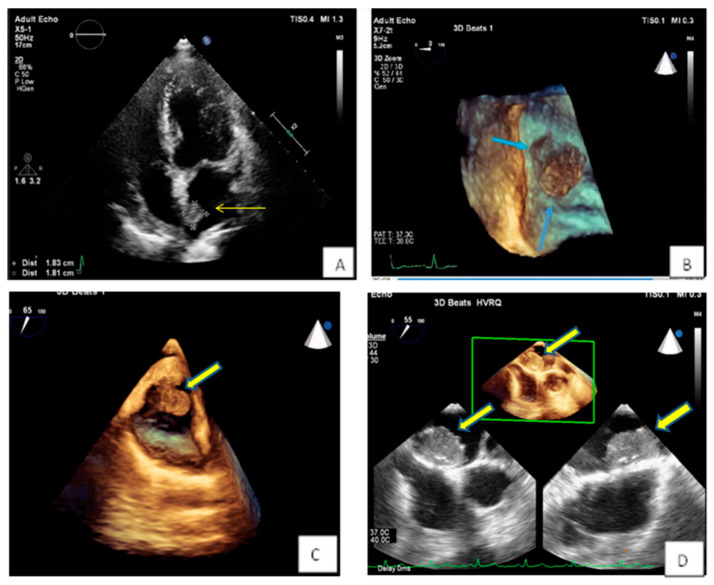
(**A**,**B**) Spherical lesion 1.8 × 1.8 cm attached through a wide base close to fossa ovalis was depicted at TTE (**A**) (yellow arrow) and subsequently TEE (**B**) (blue arrows—3D zoom). The location of the tumor in the left atrium as well as its morphological characteristics are compatible with a myxoma. (**C**) 3D TEE showing a polypoid (yellow arrow), with a smooth or mildly lobar surface located at the entrance of the inferior vena cava. Echocardiographic findings are consistent with a myxoma in a rare position. (**D**) 3D TEE depicting a large mass with spot calcific areas located at the fossa ovalis with a wide base, findings consistent with a left atrium myxoma. Please note the areas with higher echogenicity within the mass (yellow arrows).

**Figure 5:**
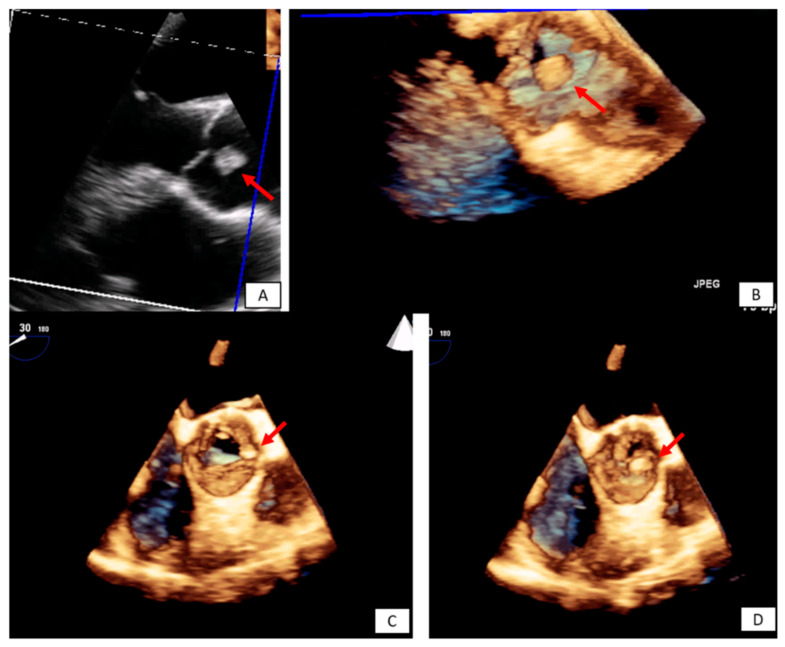
Incidental finding on TTE in an asymptomatic patient followed by TEE. The transesophageal image revealed a papillary fibroelastoma attached to the left cusp of the aortic valve (red arrows) 3D TEE with cropping in long axis (**A**,**B**), and short axis view ((**C**,**D**) during systole and diastole, respectively). Please note that the pedunculated lesion is attached on the tip of the aortic surface of the aortic valve, is not protruding in the LVOT during systole and doesn’t lead to valve destruction or insufficiency.

**Figure 6:**
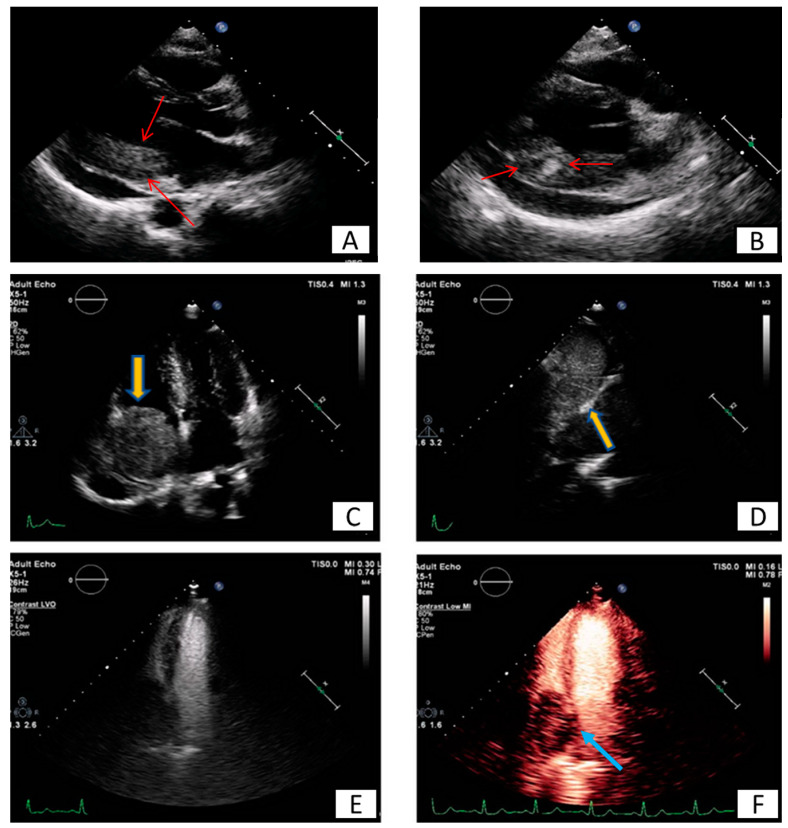
Long (**A**) and modified short axis (**B**) view of transthoracic echocardiography in a young female patient with metastatic carcinoma. Note the presence of pericardial effusion and the increased echogenicity and inhomogeneity of the basal posterior myocardial wall finding consistent with malignancy (red arrows). (**C**–**F**) Patient presenting with dyspnea on exertion and right heart failure symptoms during last months. A large mass (5.2 × 4.8 cm) is depicted inside the right atrium (**C**), originating from inferior vena cava (**D**). Utilizing echo contrast agents, the increased vasculature of the mass is revealed, a finding supportive of malignancy (**E**,**F**).

Certain cardiac tumors tend to appear in specific locations and structures [22]. Myxomas usually develop within the left atrium [23] (Figure 4). Most sarcomas also develop within the left atrium and can be mistakenly confused pre-operatively with myxomas. Angiosarcomas, on the other hand, are more often found within the right atrium [24]. Rhabdomyomas and fibromas are located in the ventricles, while papillary fibroelastomas are located on the valves [25] (Figure 5). Finally, metastatic tumors can be located anywhere in the heart, depending on the way cancer has spread [26]. In most cases masses spread through inferior vena cava to the right atrium (Figure 6).

### 3.1. Transthoracic Echocardiography

**Figure 7:**
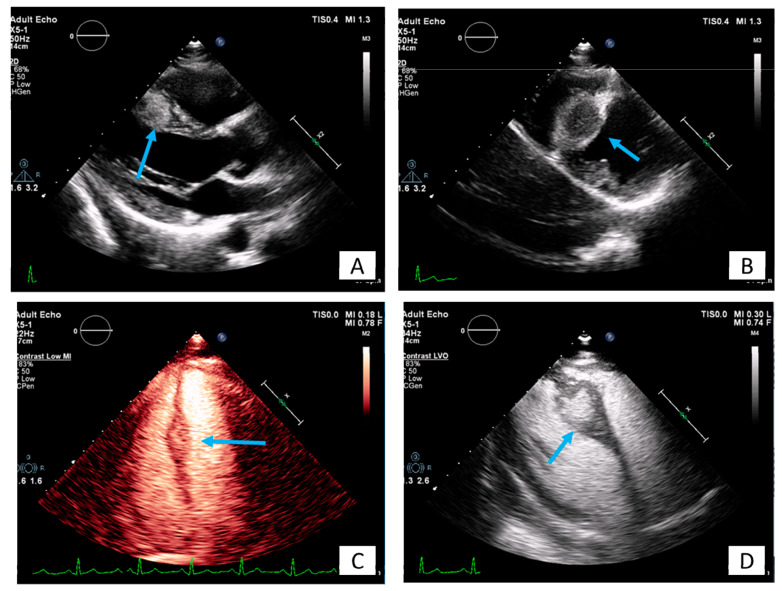
A 35-year-old patient with a round lesion located at the middle of intraventricular septum, known from 5 years ago, stable in size during follow-up (**A**,**B**). Using contrast agents, the mass is highly echogenic indicating increased vasculature with a surrounding halo (**C**,**D**). It is most likely an intramural hemangioma.

Echocardiography is simple to perform, non-invasive, widely-available, and affordable [3]. It provides accurate information on the morphology, localization, and mobility of the tumor and finally can evaluate its hemodynamic impact [27,28,29]. Transthoracic echocardiography (TTE) provides information about the echodensity and degree of calcification of the mass. Continuous Doppler velocities as well as Color Doppler can provide information on the hemodynamic effect of the mass. Furthermore, echo contrast agents (LVO or perfusion modalities) can provide significant information on morphological characteristics and tumor vascularity-perfusion [30,31,32] (Figure 7). Speckle tracking imaging can also highlight the non-contractible nature of non-mobile masses such as fibromas [31,33].

### 3.2. Transesophageal Echocardiography

**Figure 8:**
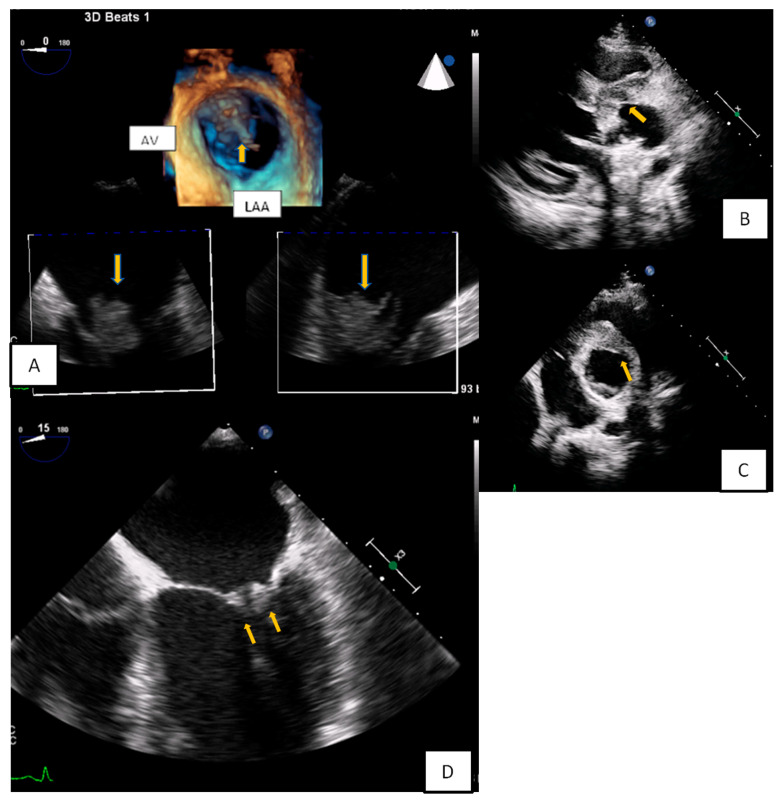
(**A**) 3D-TEE in a patient evaluated for a possible endocarditis. A large multilobular amorphous mass (vegetation) is found attached on the right commissure (3rd hour) of the anterior and posterior leaflet (AV: Aortic valve, LAA: Left atrial appendage). (**B**,**C**) A huge abscess infiltrating the aortic root and the aortomitral curtain in a young febrile patient with prosthetic aortic valve. (**D**) Transesophageal echocardiography-4 Chamber view. Increased thickness and echogenicity of both the tips of the leaflets in a patient with lupus erythromatosus endocarditis.

Transesophageal echocardiography, especially when performed utilizing three-dimensional imaging techniques, can provide a detailed anatomical study of the mass and its surrounding tissues [34,35]. TEE is superior compared to TTE and can accurately highlight the relationship between the mass and the surrounding tissue especially for atrial tumors. It is also superior to CMR when imaging small and highly mobile masses such as vegetations, papillary fibroelastomas or other masses adjacent to the cardiac valves. It is worth to point out that for tumors in the atria, TEE is of high diagnostic utility contributing to the early differential diagnosis. Furthermore, TEE may provide information for the presence of infective endocarditis’ complications such as abscess and infective infiltration of the surroundings tissues (Figure 8).

### 3.3. Computed Tomography

**Figure 9:**
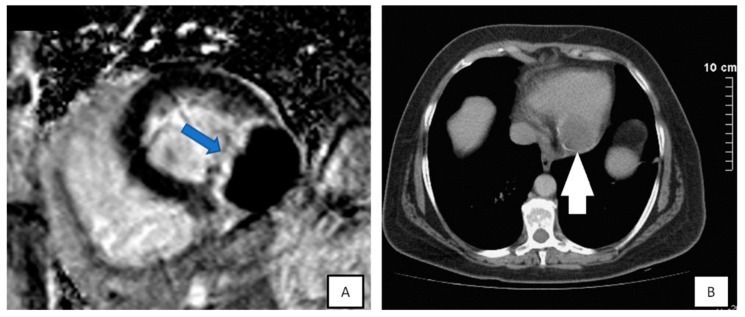
(**A**) Short-axis of late-gadolinium enhancement image demonstrating the echinococcus cyst attached to inferolateral wall, highly hypointense with hyperintense border (blue arrow). (**B**) CT demonstrated a calcified cyst (white arrow) proved to be a cardiac echinococcus cyst.

**Figure 10:**
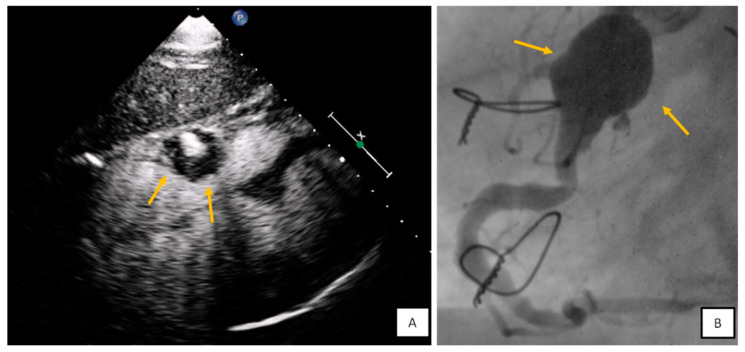
(**A**) Subxiphoid view with contrast agent. Round mass with mural thrombus and blood flow at the level of tricuspid annulus findings consistent with right coronary artery (RCA) aneurysm. (**B**) Coronary angiography confirmed the diagnosis.

CT and CMR are complementary and useful especially if surgical resection of the tumor is planned [36,37,38,39]. For both techniques acquisition protocols may vary and should be guided by clinical scenario and echocardiography data. CT can provide information about tumor vascularity (using contrast enhancement), the extent of tumor calcification, the presence of fat tissue, as well as the staging of the patient [39,40,41]. CT is the modality of choice in masses adjacent to the prosthetic valves and is superior to CMR for calcified masses (Figure 9). Compared to CMR, CT has superior spatial resolution facilitating 3D reconstruction [40,42]. Recent advances using spectral CT have also improved tissue characterization with decrease of radiation dose at the same time. Analysis of myocardial strain provides incremental value in the context of complex cardiac structures including cardiac masses. CT can evaluate the extracellular volume to identify infiltration and fibrosis [43]. CT or coronary angiography can be performed to diagnose coronary aneurysms (Figure 10).

### 3.4. Cardiac Magnetic Resonance

CMR has adequate spatial and temporal resolution, and enables multi-planar reconstruction. It has tissue characterization capabilities due to inherent soft-tissue contrast, which can be brought out by using different tissue-weighted sequences and augmented by the administration of gadolinium-based contrast agents [44]. CMR differentiates benign from malignant cardiac masses, with tumor size, heterogeneity of signal intensity, local infiltration, and enhanced initial perfusion being the most reliable indicators of malignancy [38,45,46,47]. 3D-MRA is a new sequence that diagnoses cardiac tumors and can also disclose vascular involvement. Tumor vascularization can also be assessed by other perfusion techniques (contrast echo, angiography) [30,39,45]. Hence, concerning patients with malignant tumors, CMR offers complementary information and helps in diagnosis and patient’s management.

On the other hand, it may not recognize effectively some small and highly mobile benign masses previously depicted with echocardiography [48]. CMR, when compared to CT, is superior for tissue characterization and reveals presence of fat tissue, degree of tissue edema and iron content of the cells [36,39,49]. CMR does not expose patients to ionizing radiation, but has lower spatial resolution and is inferior in detecting mass calcification. Performing a CMR study is more time consuming and need more coordinative patients. 3D reconstruction is available for both techniques and can be used for preoperative planning [42]. In infants and children, echocardiography is even more frequently used because of the limitations of CMR and cardiac CT [50]. However, when available, CMR can in most cases predict the tumor type in pediatric patients [51,52].

**Figure 11:**
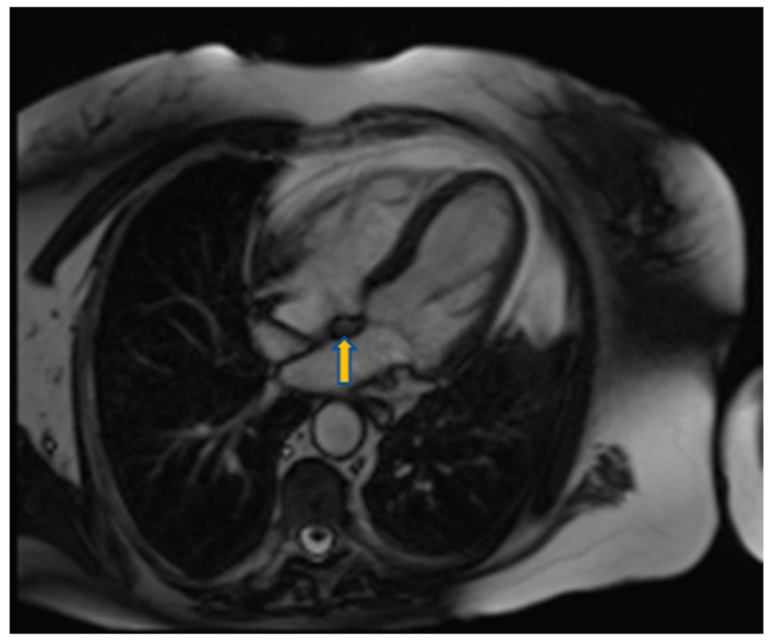
Four chamber view using SSFP sequence of a 70-year-old man showing lipomatous hypertrophy of the atrial septum (arrow).

Specific considerations related to CMR imaging include:

1. Tissue characterization on T1–T2 weighted images:
  - Imaging characteristic of hemorrhagic pericardial effusion and clots depends on the age of the effusion.
  - Contrast enhancement during the first pass T1 weighted imaging is indicative of increased vasculature.
  - T1 hypo/isointensity with high T2 weighted signal intensity characterizes the majority of benign or malignant cardiac tumors
  - Apical and hypertrophic cardiomyopathy may mimic cardiac tumors such as fibromas. In such cases fibromas appear hypointense in T2 weighted images while hypertrophic cardiomyopathy hyperintense. Moreover, CMR tagging can highlight the contractile nature of hypertrophic cardiomyopathy [53,54]. High T1 and low T2 weighted signal intensity is indicative of metastatic malignant melanoma.
  - High T1 signal intensity is also seen in lipomas. These masses and other lipomatoses can be recognized with fat suppression techniques (Figure 11).
2. Early gadolinium enhancement (EGE) which identifies thrombus from the adjacent hypokinetic cardiac wall.
3. Late gadolinium enhancement (LGE) that is ideal for fibrosis and necrosis identification.

### 3.5. Positron Emission Tomography

Furthermore,^18^F FDG-PET provides valuable information on the metabolic activity of cardiac tumors, differentiates benign from malignant cardiac tumors and predicts survival [7,55,56]. When there is no ^18^F-FDG uptake in a lesion, malignancy can be excluded in most cases. False-positive ^18^F-FDG uptake can be seen in inadequate patient preparation, inflammatory conditions (e.g., sarcoidosis), infections, abscesses, surgical changes, radiation changes, and brown fat. Other hybrid modalities (PET/MRI) have further incremental value [56,57]. Hence, in cases that the malignant nature of the cardiac mass is still under question, PET/CT or PET/MRI can be used to increase the diagnostic accuracy and/or to define the staging of the patient with malignancy, so that oncology team can propose and initiate further medical care.

## 4. Conclusions

**Figure 12:**
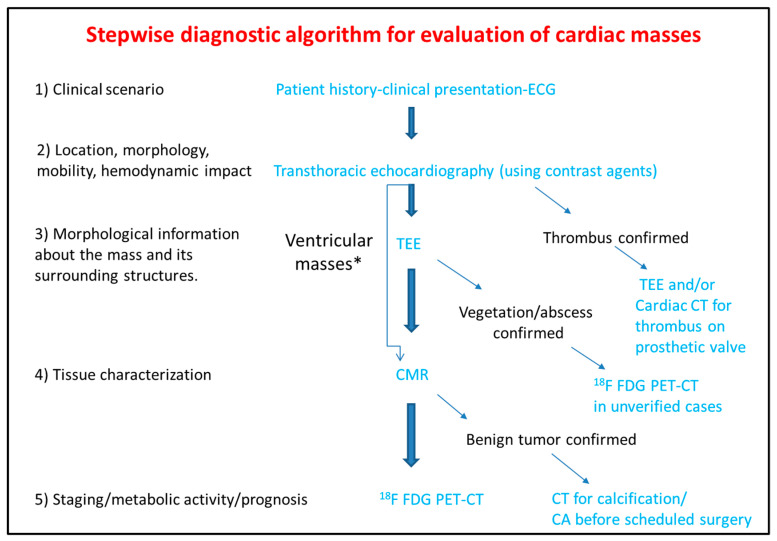
Step-by-step diagnostic algorithm for cardiac masses using cardiovascular imaging. Patient’s history, clinical data and ECG should be acquired and evaluated before extensive imaging work-up. With data extracted by the clinical evaluation and the TTE, a physician may initiate differential diagnosis and plan further work-up approach. When the mass is proved to be a thrombus (consider using ultrasound enhancing agents) no further work-up is needed. When thrombus is found on a prosthetic valve, TEE and/or CT can also be used. The next step is TEE especially for atrial masses. When the mass is vegetation or abscess then usually no-further work up is needed (consider PET scan as next best step when diagnosis is still under question). For other masses CMR is the next best step and guides further work-up which could include CT, or ^18^F FDG * For ventricular masses which are not adjacent to the cardiac valves and are not highly mobile, CMR can be alternatively used after the transthoracic echocardiogram without the need of TEE.

The differential diagnosis of the cardiac masses is critical for the clinical management of the patient. Patient history and clinical examination are evaluated in combination with cardiovascular imaging data. Experience when performing TTE or TEE and application of newer echocardiographic techniques such as contrast echocardiography, usually set the initial diagnosis and guide further work-up with the use of CT, CMR, and ^18^F FDG PET-CT. The latter offer additional incremental information regarding diagnosis, therapeutic management and prognosis (Figure 12).

PET-CT.ECG: Electrocardiogram; TEE: Transesophageal echocardiogram; CMR: Cardiac magnetic resonance; CT: Computed tomography; ^18^F FDG PET: Fluorodeoxyglycose positron emission tomography; CA: Coronary angiography.

## Figures and Tables

**Table 1:** Clinical, echocardiographic and cardiac magnetic resonance (CMR) characteristics of cardiac masses.

	Common Age at Presentation	Common Location at Heart	Clinical Manifestations	Echocardiography	CT	CMR
Benign primary heart tumors						
Myxoma	Early (familial) or middle adulthood	LA, atrial septum, any other site	Emboli, flow obstruction, systemic symptoms	Mildly lobar, heterogeneous echodensity, usually mobile (with or without stalk)	Heterogeneous, low attenuation, may be calcified	Isointense T1w, High T2w, heterogeneous LGE
Papillary fibroelastoma	Middle or late adulthood	Cardiac valves	Usually asymptomatic, emboli	Circular, pedunculated, non-protruding, usually not causing valve dysfunction	Smooth, pedunculated	Iso/hypointense T1w, high T2w, LGE, highly mobile, consider TEE
Lipoma	Adulthood	Left ventricle, any other site	Usually asymptomatic, arrhythmias, flow obstruction	Homogeneous	Smooth, homogenous encapsulated, fat attenuation, no enhancement with contrast	High T1w, T2w, no LGE, suppressed with SPIR
Rhabdomyoma	Infancy or early childhood	Ventricles, atrioventricular valves	Usually asymptomatic, flow obstruction, heart failure, arrhythmias	Homogenous, slightly echogenic, can be multiple	Attenuation similar to myocardium, intramural	Isointense T1w, iso/hyperintense T2w, no LGE
Fibroma	Early childhood	Intraventricular septum, ventricles	Usually asymptomatic, arrhythmias	Heterogeneous, echogenic, non-contractible, can mimic HCM	Soft tissue attenuation, low contrast enhancement, may be calcified	Isointense T1w, Low T2w, high LGE
Hemangioma	Any age	Any other site	Usually asymptomatic, dyspnoea	Highly echogenic with contrast infusion, may resemble a cavity	Heterogeneous, high contrast enhancement, may be calcified	Heterogeneous, high T1w, Very high T2w, centripetal progression on first pass, heterogeneous LGE,
Malignant primary heart tumors						
Angiosarcoma	Early and middle adulthood	RA, pericardium	Constitutional symptoms, heart failure, pericardial effusion	Heterogeneous, highly echogenic with contrast infusion	Heterogeneous, irregular, low attenuation	Heterogeneous T1w, T2w LGE, early enhancement at first pass
Rhabomyosarcoma	Childhood, early adulthood	Ventricles, any other site	Heart failure	Normal-high echodensity	Irregular, low attenuation	Isointense T1w, high T2w, usually homogenous LGE
UPS/Myxofibrosarcoma	Early and middle adulthood	Left atrium, any other site	Flow obstruction/heart failure, pericardial effusion, metastatic	Heterogeneous, normal-high echodensity	Heterogeneous, low attenuation	Heterogeneous T1w, T2w LGE
Lymphoma	Adulthood	RA, any other site	Pericardial effusion, flow obstruction/heart failure	Any size and shape	Normal to low attenuation, heterogeneous contrast enhacncement	Isointentse T1w, T2w, minimal LGE
Mesothelioma	Adulthood	Pericardium	Pericardial effusion	Heterogeneous	Variable characteristics	Isointense T1w, Heterogeneous T2w, high LGE
Metastatic tumors						
Melanoma	Adulthood	Any site	Flow obstruction/heart failure, pericardial effusion	Heterogeneous, highly echogenic with contrast infusion	Similar to soft tissue, hard to recognize	High T1w, low T2w heterogeneous LGE
Other metastatic tumors	Adulthood	Pericardium, any site	Flow obstruction/heart failure, pericardial effusion	Heterogeneous, highly echogenic with contrast infusion	Solid, similar to soft tissue attenuation, moderate to high contrast enhancement	Low T1w, high T2w, heterogeneous LGE
Non-neoplasmatic heart masses						
Clots	Adulthood	LAA, LV apex	Emboli	Acute: Low echodensity Chronic: High echodensity No perfusion with contrast agents	No contrast enhancement, may be calcified	No EGE/LGE Acute: Isointense to high T1w, T2w Subacute: High T1w, Low T2w Chronic: Low T1w, T2w
Vegetation	Adulthood	Cardiac valves	Valve dysfunction, emboli, heart failure	Highly mobile, oscillating protruding, valve dysfunction,	Low attenuation, may recognize, perivalvular extension, fistulas, abscess	Highly mobile, consider TEE
Non-neoplasmatic calcified masses	Adulthood	Usually posterior mitral annulus	Usually asymptomatic	Very high echodensity	Heterogeneous, calcified	Signal loss, consider CT
Pericardial cysts	Adulthood	Pericardium	Usually asymptomatic, external compression	Low echodensity, typically no perfusion with contrast agents	Presence of wall, fluid attenuation	Depends on the fluid, usually isointense T1w, high T2w, no LGE
Lipomatous Hypertrophy	Late adulthood	Atrial septum	Usually asymptomatic, arrhythmias	Homogeneous dumbbell appearance of atrial septum	Fat attenuation	High T1w, T2w, no LGE, suppressed with SPIR

CMR: Cardiac magnetic resonance; CT: Computed tomography; EGE: Early gadolinium enhancement; HCM: Hypertrophic cardiomyopathy; LA: Left atrium; LAA: Left atrium appendage; LGE: Late gadolinium enhancement; LV: Left ventricle; RA: Right atrium; SPIR: Spectral presaturation with inversion recovery; TEE: Transesophageal echocardiography; UPS: Undifferentiated pleomorphic sarcoma.
